# Early cortico-muscular coherence and cortical network changes in Parkinson’s patients treated with MRgFUS

**DOI:** 10.3389/fneur.2024.1362712

**Published:** 2024-03-22

**Authors:** Elisa Visani, Ferruccio Panzica, Silvana Franceschetti, Nico Golfrè Andreasi, Roberto Cilia, Sara Rinaldo, Davide Rossi Sebastiano, Paola Lanteri, Roberto Eleopra

**Affiliations:** ^1^Epilepsy Unit, Fondazione IRCCS Istituto Neurologico Carlo Besta, Milan, Italy; ^2^Clinical Engineering, Fondazione IRCCS Istituto Neurologico Carlo Besta, Milan, Italy; ^3^Neurophysiopathology Unit, Fondazione IRCCS Istituto Neurologico Carlo Besta, Milan, Italy; ^4^Parkinson and Movement Disorders Unit, Fondazione IRCCS Istituto Neurologico Carlo Besta, Milan, Italy; ^5^Functional Neurosurgery Unit, Fondazione IRCCS Istituto Neurologico Carlo Besta, Milan, Italy

**Keywords:** Parkinson’s disease, cortico-muscular coherence, cortical network, MRgFUS, MEG

## Abstract

**Introduction:**

To investigate cortical network changes using Magnetoencephalography (MEG) signals in Parkinson’s disease (PD) patients undergoing Magnetic Resonance-guided Focused Ultrasound (MRgFUS) thalamotomy.

**Methods:**

We evaluated the MEG signals in 16 PD patients with drug-refractory tremor before and after 12-month from MRgFUS unilateral lesion of the ventralis intermediate nucleus (Vim) of the thalamus contralateral to the most affected body side. We recorded patients 24 h before (T0) and 24 h after MRgFUS (T1). We analyzed signal epochs recorded at rest and during the isometric extension of the hand contralateral to thalamotomy. We evaluated cortico-muscular coherence (CMC), the out-strength index from non-primary motor areas to the pre-central area and connectivity indexes, using generalized partial directed coherence. Statistical analysis was performed using RMANOVA and *post hoct*-tests.

**Results:**

Most changes found at T1 compared to T0 occurred in the beta band and included: (1) a re-adjustment of CMC distribution; (2) a reduced out-strength from non-primary motor areas toward the precentral area; (3) strongly reduced clustering coefficient values. These differences mainly occurred during motor activation and with few statistically significant changes at rest. Correlation analysis showed significant relationships between changes of out-strength and clustering coefficient in non-primary motor areas and the changes in clinical scores.

**Discussion:**

One day after MRgFUS thalamotomy, PD patients showed a topographically reordered CMC and decreased cortico-cortical flow, together with a reduced local connection between different nodes. These findings suggest that the reordered cortico-muscular and cortical-networks in the beta band may represent an early physiological readjustment related to MRgFUS Vim lesion.

## 1 Introduction

In Parkinson’s disease (PD), resting tremor is a cardinal feature that primarily supports the early diagnosis (1). In addition, postural and kinetic tremors are also common manifestations. In several patients, tremor occurs unilaterally, namely in the relatively early disease stages (2). Tremors can occur in the earliest disease stages, and studies using magnetoencephalography (MEG) have shown that oscillatory activity in the motor cortex, cerebellum, and diencephalic area are tremor-related (3). Cerebello-thalamocortical circuit, the basal ganglia, and the interaction between these two circuits are primarily implicated in the generation of all symptoms (4).

Cortical structures are certainly strongly involved in the disorder, including the motor cortex. Substantia nigra dopaminergic neurons influence the firing rate and synchronization of motor cortical neurons through direct projections and indirect pathways involving the basal ganglia and motor thalamus. Moreover, in PD pathophysiology, the motor cortex is responsible for transferring abnormal activity occurring in the basal ganglia to muscles (5), and is the basis of a positive effect of transcranial magnetic stimulation in PD (6). Moreover, long-range input to the motor cortex originating from other cortical areas may play a role in various movement disorders, including PD (7). These include the primary somatosensory cortex, the contralateral motor cortex, secondary motor cortices [premotor cortex and supplementary motor cortex demonstrated in primates (8, 9)] and other frontal regions (10). These inputs are also probably involved in the side effects of levodopa in PD patients (11).

Among different treatments, surgical options, such as deep brain stimulation and magnetic resonance-guided focused ultrasound (MRgFUS) thalamotomy (12, 13), are a part of the therapeutic opportunities in selected patients, presenting with prominent tremors, as well as in other pathological conditions with tremors, such as essential tremor (14).

We analyzed MEG signals that non-invasively and directly measures the magnetic fields generated by neuronal activity of the cerebral cortex with high spatial and temporal resolution. MEG signals are not distorted by the skull, scalp, and require a simpler head model to apply for source localization. This feature makes MEG a valuable tool that can be used to investigate and disentangle the complex interactions of neural populations, or to localize the physiological and pathological activities.

We are reporting here information concerning the neocortical reorganization involving the motor cortex and non-primary motor cortical areas detected on neurophysiological (MEG) signals in patients with prominently unilateral tremors treated with unilateral VIM thalamotomy using MRgFUS in the absence of other severe symptoms.

## 2 Materials and methods

### 2.1 Subjects

We included 16 patients (Table 1) diagnosed with clinically probable PD (1) and tremor-dominant motor phenotype, who showed a clearly prominent tremor on an upper arm and who were followed up for more than 1 year.

**Table 1:** Demographics, clinical scores and adverse events at baseline and 12-months follow-up.

Demographic characteristics		
Sex (M/F)	13/3	
Age (years)	67.1 ± 8.4	
Age at onset (years)	60.5 ± 6.9	
Disease duration (years)	6.7 ± 3.8	
Treated side (left/right thalamus)	8/8	
**Baseline MDS-UPDRS-I, median [IQR]**		
Total score	2.5 [1.75; 5.3]	
Item 1.1 Cognitive impairment	0 [0; 1]	
Item 1.2 Hallucinations and psychosis	0 [0; 0]	
Item 1.3 Depressed mood	0 [0; 1]	
Item 1.4 Anxious mood	0 [0; 1]	
Item 1.5 Apathy	0 [0; 0]	
Item 1.6 Features of dopamine dysregulation syndrome	0 [0; 0]	
**Motor Outcome (MDS-UPDRS-III ON medication)**		
	Baseline	*12 months*
Total score	27.6 ± 9.7^**^	20.3 ± 9.6^**^
Tremor score^a^	6.1 ± 1.9^***^	1.6 ± 1.8^***^
Bradykinesia score^b^	5.1 ± 2.7	4.4 ± 3.1
Rigidity score^c^	2.3 ± 1.0^***^	0.8 ± 1.1^***^
Axial score^d^	4.3 ± 2.0^*^	5.4 ± 2.9^*^
H&Y, median (IQR) [min-max]	2 (2; 2) [1–2]	2 (2; 2) [2–2]
**Pharmacological therapy**		
LEDD^e^ (mg), mean ± SD (min-max)	570 ± 329 (0–1,350)	572 ± 244 (150–1,000)
Anticholinergic, *n* (%)	3 (19%)	2 (13%)
Beta-blocker, *n* (%)	1 (6%)	1 (6%)
**Thalamotomy related adverse events**		
Patients with 1 or more AE (*n*)	11	2
Type of AE		
Gait imbalance (*n*)	8	0
Perioral/hand paresthesia (*n*)	6	2
Dysarthria (*n*)	4	0
Inferior limb weakness (*n*)	2	0
Facial asymmetry (*n*)	1	0

Data expressed as mean ± SD unless otherwise specified. AE, Adverse Event; H&Y, Hoehn and Yahr stage; IQR, Interquartile range; LEDD, Levodopa Equivalent Daily Dose, MDS-UPDRS-I Movement Disorder’s Society Unified Parkinson’s Disease Rating Scale Part I, Non-Motor Aspects of Experiences of Daily Living; MDS-UPDRS-III, Movement Disorder’s Society Unified Parkinson’s Disease Rating Scale Part III, Motor score. ^*^p < 0.05; ^**^p < 0.010; ^***^p < 0.001. ^a^Sum of items 3.15, 3.16, 3.17 relative to the treated side; ^b^Sum of items 3.4–3.8 relative to the treated side; ^c^Sum of items 3.3 of upper and lower limbs relative to the treated side; ^d^Sum of items 3.1, 3.2, 3.9–3.13; ^e^LEDD calculated as previously described (15, 16); LEDD of safinamide was calculated as described in Cilia et al. (17).

The majority of patients were male (13), but there were no obvious differences in both demographic data (age: males = 66.8 ± 2.3 years; females = 68.7 ± 1.7 years; onset age: males = 59.9 ± 1.9 years, females = 63.0 ± 1.5 years) and data obtained from both clinical and neurophysiological measures.

MDS-UPDRS scores were assessed 24 h before MRgFUS (T0), 24 h after the procedure (T1) and at 12-months follow-up. Detailed pharmacological therapy has been recorded; Levodopa-Equivalent Daily Dose (LEDD), was calculated as previously reported (15, 16), LEDD of safinamide was calculated as recently reported (17) (Table 1). The tremor was scored using the different components of the International Parkinson and Movement Disorders Society version of the Unified Parkinson’s Disease Rating Scale (MDS-UPDRS) (18, 19). MDS-UPDRS scores were assessed 24 h before MRgFUS (T0), 24 h after the procedure (T1) and at 12-months follow-up.

We considered eligible for MRgFUS treatment patients with tremor refractory to drug therapy; other significant pathologies or medical risk factors were considered as exclusion criteria, including the presence of cognitive decline and significant psychiatric comorbidities. Detailed MRgFUS eligibility criteria have been previously reported (20). Eight patients underwent MRgFUS treatment on the left thalamus and eight on the right thalamus.

At the time of our evaluation, all patients were on their current home medication regimen and in the “medication-on” condition.

MDS-UPDRS-I and MDS-UPDRS-III were evaluated at T0 and after 1 year of follow-up. The sub scores for tremor (items 3.15, 3.16, 3.17) rigidity (sum of items 3.3 of upper and lower limbs) and bradykinesia (items 3.4–3.8) relative to the treated side and axial score (items 3.1, 3.2, 3.9–3.13) were computed. Only the MDS-UPDRS-III tremor score was evaluated at T1.

We chose the medication-on condition as this was more similar to the daily condition of the patients and avoided an uncomfortable situation during the tests carried out during the MEG recording.

At T1, 11 patient presented symptoms referable to minimal adverse events in the immediate post-treatment, which completely recovered in nine and mitigated in in two (Table 1).

The study was approved by the Ethics Committee of the Fondazione IRCCS Istituto Neurologico C. Besta and was carried out according to the Declaration of Helsinki, and its amendments. All subjects provided their written informed consent before being included in the study.

### 2.2 MEG signals acquisition and analysis

MEG signals were recorded with a whole-head system (Neuromag Triux, MEGIN; Finland) and pre-processed according to our laboratory procedures [see (21), for details]. For the analyses, we selected an epoch of 60 s at rest and epochs of the MEG and concomitant EMG signals during repeated isometric extensions of the hand contralateral to the ViM target. To limit the presence of tremor that usually appears in the stationary phase of isometric contraction, we selected multiple epochs at the start of each extension (reaching an analysis time of 60 s). Source time series were extracted with a linearly constraint minimum variance beamforming approach using a head model based on individual MRI. Data were normalized to the MNI template to extract the source time series on different cortical areas according to the Automated Anatomical Labeling atlas. The included regions of interest (ROIs) were: Precentral (PreC), Postcentral (PostC), Supplementary Motor (SupM), Parietal (P, including inferior and superior parietal areas), and Frontal (F, including superior and middle gyri) ROI of contralateral (Co) hemisphere with respect to the activated hand. The mean of the values measured on the same ROIs in the ipsilateral hemisphere were grouped into an ROI called Ipsi. We analyzed the MEG signals in different frequency bands: delta (0–4 Hz), theta (>4–8 Hz) alpha (>8–13 Hz), beta (>13–30 Hz), low-gamma (>30–45 Hz).

Cortico-muscular coherence (CMC) between cortical ROIs and muscular activity during isometric contraction was estimated at T0 and T1 using a block-wise bivariate autoregressive parametric model. The CMC values were normalized (nCMC) to the maximum value obtained at different times to better highlighting the coherence reorganization in the different cortical ROIs. To investigate cortical connectivity, generalized partial directed coherence [gPDC, (22)] was applied to the same epochs selected to estimate the nCMC and in the epoch at rest. For CMC and gPDC methods see our previous studies in patients with cortical myoclonus (23–26) and in a population of patients with essential tremor treated with MRgFUS (27). To investigate the regional properties of the network and the unidirectional coupling between ROIs, we calculated the out-degrees (number of edges going out of a node, considering each ROI as a node) and the out-strength index (edges values), respectively. Moreover, we calculated the betweenness centrality, measuring the centrality in a graph of a specific region based on shortest paths, and the clustering coefficient, measuring the degree to which a network organizes into a region. Data analysis was performed using custom-made Matlab (MATLAB 2016a, Mathworks, Inc., Natick, MA, United States) scripts based on the Fieldtrip toolbox (28).

### 2.3 Statistical analysis

nCMC, out-strength measures, and connectivity indexes obtained in selected cortical ROIs were compared using repeated measures ANOVA (RM ANOVA) at a significance level of *p* < 0.05, using ROIs and Time (T0, T1) as the within-group factor. The sphericity assumption was evaluated using Mauchley’s test, and the Greenhouse–Geisser degree of freedom correction was applied when appropriate. Where the RM ANOVA indicates a significant factor or interaction, post-hoc tests using independent and paired samples were performed. Values are expressed as mean ± standard error of the mean.

To test the relationship between clinical scores evaluated at 12-months follow-up and neurophysiological measures linear regression was applied.

## 3 Results

### 3.1 Cortico-muscular coherence

At T0, CMC showed a peak in the alpha band in 12 out of 16 patients with a frequency ranging from 10.4 and 11.2 Hz, in the different ROIs. The same occurred at T1, even if in eight patients only. In the delta and theta bands, no patient had detectable CMC peaks at both T0 and T1, while in the low-gamma (30–50 Hz) bands small peaks had variable CMC values and did not show significant differences between T0 and T1.

At T0, in the beta band, six patients showed a CMC peak in one or more of the selected ROIs contralateral with respect to the activated hand, while at T1 all patients but one showed a CMC peak in one or more Co-ROIs (mean frequency at T0: 23.7 ± 0.4 Hz; at T1: 21.1 ± 1.4 Hz). The frequency did not differ between T0 and T1.

**Figure 1:**
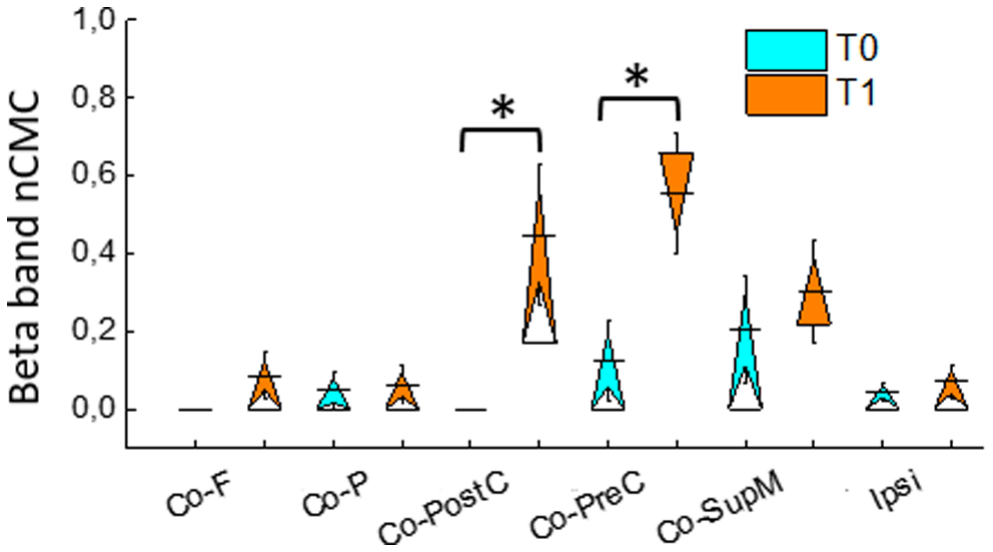
Normalized cortico-muscular coherence (nCMC) in the beta band, evaluated during isometric contraction, in different ROIs contralateral (Co) to activated hand (Co-PreC, precentral; Co-PostC, postcentral; Co-SupM, supplementary motor; Co-P, parietal; Co-F, frontal) and in the ipsilateral ROI. Asterisks indicate significant differences between the values assessed at T1 and T0.

Since CMC, normalized to its main peak (nCMC), was almost exclusively found in the ROIs directly involved motor function, and very rarely in other ROIs, including those of the hemisphere ipsilateral with respect the MRgFUS treatment, the RMANOVA was performed on the beta nCMC values including Co-PreC, Co-PostC, and Co-SupM ROIs. There were significant within-subjects effects for ROIs [*F*(3,42) = 7.8, *p* = 0.001], Time [*F*(3,42) = 8.8, *p* = 0.005] and interaction ROI x Time [*F*(3,42) = 9.3, *p* = 0.001; Figure 1].

*Post hoc* analyses revealed that, comparing the values recorded at T1 with those recorded at T0, a significant increase in nCMC value occurred in Co-PostC [*t*(15) = 3.6, *p* = 0.002] and Co-PreC ROIs [*t*(15) = 3.8, *p* = 0.002].

### 3.2 Cortico-cortical out-strength

With the aim of further exploring changes occurring at T1 with respect to T0 in the primary motor cortex, we analyzed the out-strength from Co and Ipsi ROIs toward the Co-PreC ROIs.

During isometric hand extension (action), RMANOVA showed significant within-subjects effects of ROIs but not for Time in theta, alpha, beta, and low-gamma bands. Only in the beta band, RMANOVA revealed a significant within-subjects effect of ROIs [*F*(2.23, 33.5) = 19.37, *p* < 0.001], Time [*F*(1, 15) = 29.63, *p* < 0.001] and ROIs × time *F*(4,60) = 4.29, *p* = 0.004.

**Figure 2:**
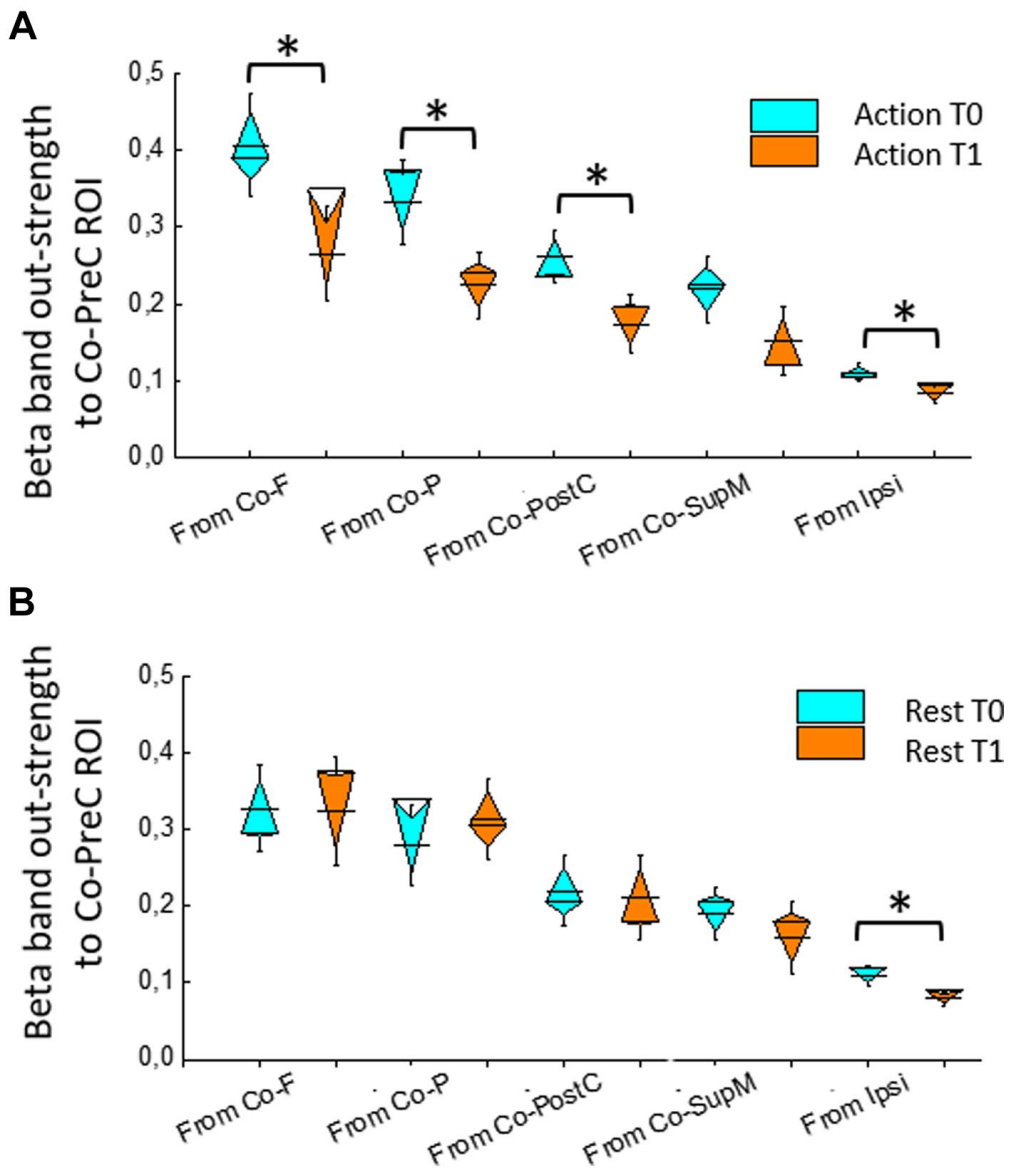
Beta band out-strength toward precentral ROI (Co-PreC) from other ROIs (Co-PreC, precentral; Co-PostC, postcentral; Co-SupM, supplementary motor; P, Co-parietal; F, Co frontal) and in the ipsilateral (Ipsi) ROI evaluated during isometric hand extension **(A)** and at rest **(B)**. Asterisks indicate significant differences between T1 and T0.

Comparing T1 and T0, a reduced out-strength toward Co-PreC ROI occurred from Co-F [*t* = 2.9(15), *p* = 0.011], Co-P [*t*(15) = 3.7, *p* = 0.002], Co-PostC [*t*(15) = 3.1, *p* = 0.007], and Ipsi ROIs [*t*(15) = 2.8, *p* = 0.012; Figure 2A].

Moreover, the out-strength from the hemisphere ipsilateral with respect the MRgFUS treatment was obviously decreased on the ROIs more involved in motor function [*t*(15) = 2.9, *p* = 0.011], while was at the significance limits from the analyzed F and P ROIs [*t*(15) = 2.1, *p* = 0.047].

When analyzing the epochs at rest, in the beta band, RMANOVA also found a significant within-subjects effect of ROIs, but without the effect of the Time (Figure 2B).

### 3.3 Connectivity indexes

For the out-degrees, during hand extension, RMANOVA showed significant within-subjects effects of ROIs in the alpha [*F*(2.53,38.03) = 5.72, *p* = 0.004], beta [*F*(1.65,24.73) = 225.141, *p* < 0.001], and low-gamma bands [*F*(2.14,32.17) = 23.42, *p* < 0.001], but no effects of Time. No effects were found for in-degrees, while betweenness centrality showed a within-subject effect of ROIs in the beta band only [*F*(3.36, 50.47) = 3.45, *p* = 0.020], but no effects of Time.

For clustering coefficient, during hand extension, in the beta and low-gamma bands RMANOVA showed a significant within-subjects effect of ROIs [beta: *F*(5,75) = 7.14, *p* < 0.001; low-gamma: *F*(2.84,42.64) = 5.17, *p* = 0.004] and Time [beta: *F*(1,15) = 26.26, *p* < 0.001]; low-gamma: [*F*(1,15) = 6.97, *p* = 0.019]. When examining the clustering coefficient in resting condition, RMANOVA did not show significant differences except for the effect of ROIs in low-gamma band [*F*(5,75) = 10.41, *p* = 0.001].

**Figure 3:**
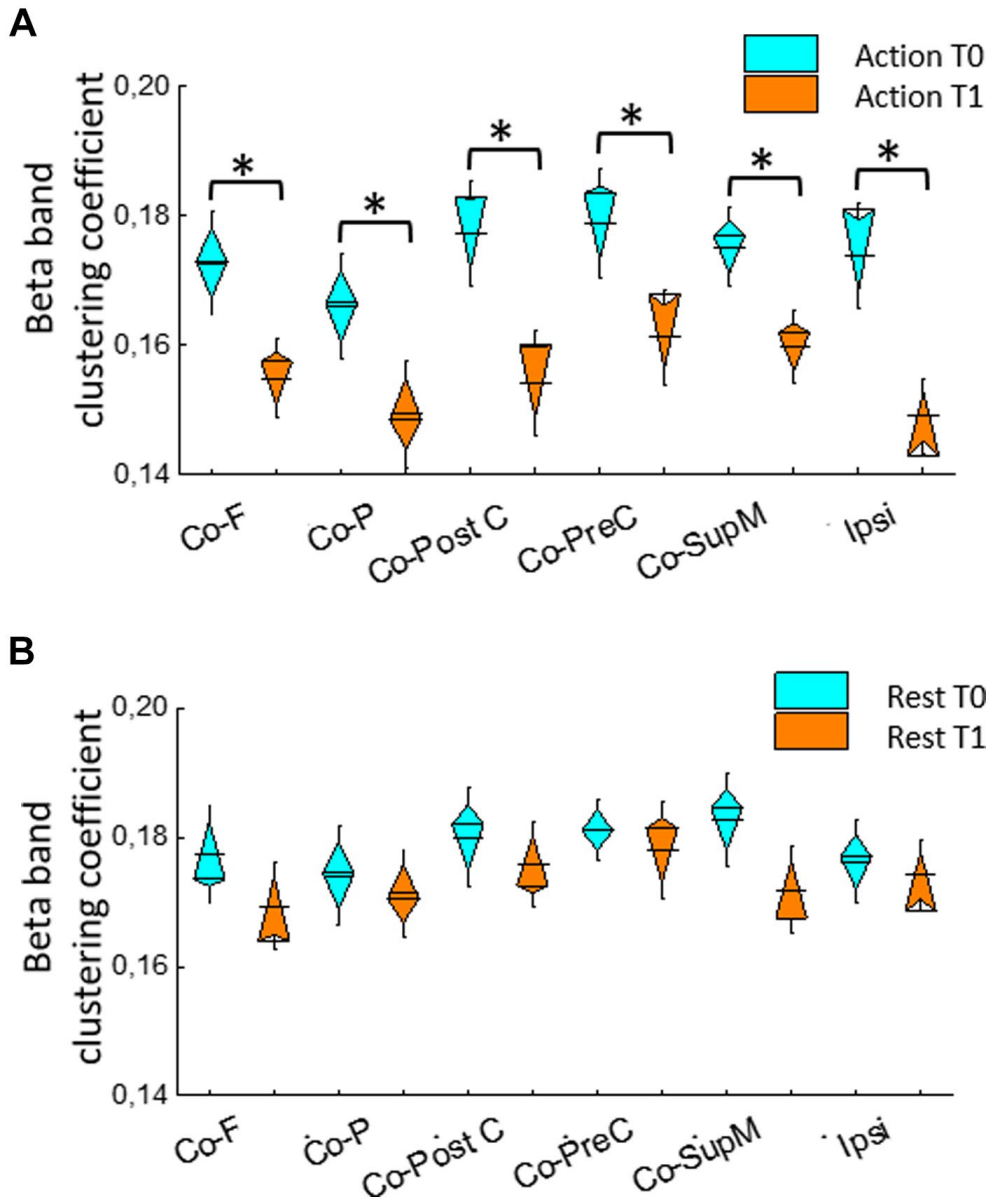
Beta band clustering coefficient in different ROIs contralateral to activated hand (Co-PreC, precentral; Co-PostC, postcentral; Co-SupM, supplementary motor; Co-P, parietal; Co-F, frontal) and in the ipsilateral (Ipsi) ROI evaluated during isometric extension of the hand contralateral to treated ViM **(A)** and at rest **(B)**. Asterisks indicate significant differences between T1 and T0.

Comparing T1 and T0 during isometric hand extension, the clustering coefficient decreased in all ROIs including the group of ipsilateral ROIs (with t value ranging from 2.7 to 6.7 and *p* values ranging from 0.037 to <0.001; Figure 3A).

When grouped together, in the Co ROIs more involved in the motor function, the clustering coefficient decreased in the beta band [*t*(15) = 4.8, *p* < 0.001], as well it decreased in symmetric ipsilateral ROIs [*t*(15) = 3.6, *p* = 0.001]. A similar decrease was found in Co [*t*(15) = 2.8, *p* = 0.008] and ipsilateral (2.7, *p* = 0.011) F ROIs and in Co [*t*(15) = 1.6, *p* = 0.011; *t*(15) = 2.6, *p* = 0.012] and ipsilateral [*t*(15) = 2.9, *p* = 0.006] P ROIs.

In the low-gamma band, the clustering coefficient decreased in Co ROIs more involved in the motor function [*t*(15) = 4.3, *p* < 0.001] and in the same ipsilateral ROIs [*t*(15) = 2.7, *p* = 0.009]; it also decreased in Co [*t*(15) = 2.9, *p* = 0.006] and Ipsilateral [*t*(15) = 2.7, *p* = 0.006] F ROIs, but not significantly in parietal ROIs.

At rest, there was a trend toward reduced value measured a T1, but no difference reached a statistical significance (Figure 3B).

### 3.4 Correlations between clinical scores and neurophysiological measures

Thirteen patients had a reduction of the total score higher than 30%, while three had a lower reduction.

**Figure 4:**
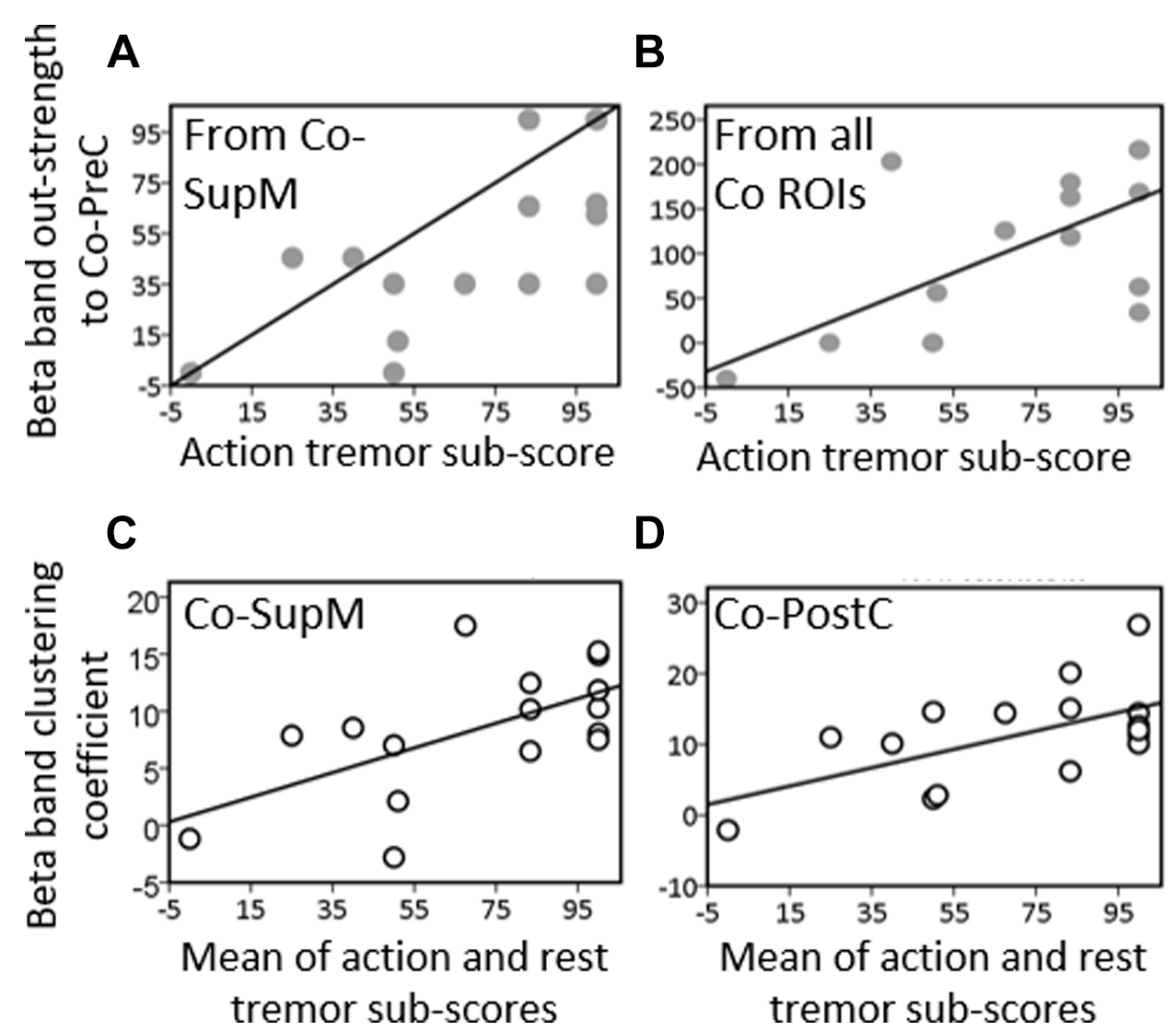
Linear regression performed between mean values action tremor sub-scores items and the values of out-strength from **(A)** contralateral supplementary motor ROI (Co-SupM), and **(B)** the sum of the out-strength values from all ROIs toward precentral area contralateral to evaluated hand. Linear regression between mean values of resting and action tremor sub-scores items and **(C)** the values of cluster coefficient on contralateral supplementary motor (Co-SupM) and **(D)** on postcentral (Co-PostC) ROIs.

Linear regression analysis showed a significant relationship between out-strength from the Co-SupM toward Co-PreC ROI [*F*(15) = 13.5, *p* = 0.002] as well as between the collective out-strength from all Co-ROIs and the percentage reduction of the tremors score during hand movement [*F*(15) = 9.7, *p* = 0.008, Figures 4A,B].

A significant relationship was also found between the clustering coefficient and the percentage reduction of the values of the mean score of tremor measured at rest and during motor activation in Co-SupM ROI [*F*(15) = 8.3, *p* = 0.012] and in the Co-PostC ROI [*F*(15) = 6.0, *p* = 0.028; Figures 4C,D].

## 4 Discussion

Dysfunction of the cerebellum-thalamocortical network and connections to other brain areas is pivotal to many types of tremors. We are reporting here information concerning the cortical reorganization detected on neurophysiological (MEG) signals after unilateral MRgFUS in patients with contralateral tremor in the absence of other severe or prominent symptom in PD patients with a 1 year of post-intervention follow-up.

In the included patients, tremor was the prominent and most disabling symptom and the VIM was selected as target. VIM is a key hub in the cerebello-thalamo-cortical circuit, which has been shown to be impaired in tremor dominant PD (29). This target is the most frequently studied in both functional neurosurgery and lesional approaches in order to treat drug-resistant tremor (30, 31). However, pathophysiology of tremor in PD is complex and involves structures of the basal ganglia-cortical loop (29). In patients with tremor accompanied by other disabling motor symptoms, other targets have been explored and demonstrated efficacy in improving not only tremor but also rigidity, bradykinesia and axial features (32).

Our main evidence concerns the readjustment of a basic mechanism (expressed through cortico-muscular coherence) connecting cortical function with the activated hand, the rearrangement of the cortico-cortical flow toward the precentral area contralateral to lateralized tremor, and the reduction affecting indexes of local cortical trafficking, expressed by clustering coefficient.

The detected changes and the resulting differences were observed the day after the MRgFUS treatment, when the tremor was absent, suggesting that the reordering of cortico-muscular coherence and cortical network in the beta band may represent a very early physiological readjustment of cortico-muscular and cortical relationships. This mainly occurred in the beta band, representing the frequency commonly associated with motor activity (33). The maintenance, in most patients, of the tremor relief 1 year after the MRgFUS treatment and the positive relationship found between changes in tremor scores and neurophysiological parameter assessed at T1 suggest that the early network reordering may also serve as predictors of late outcome.

CMC in the beta band gives information about functional coupling between muscles and the cortex (33). Beta-band CMC became evident when healthy subjects perform isometric contraction and it has been already found reduced in PD patients as a revealing factor of their motor impairment (34). Conversely, it increases after motor improvement in the presence of deep brain stimulation (35) as well as a consequence of effective levodopa treatment (36). We previously found a similar increase at T1 of beta-CMC in patients with essential tremor submitted to MRgFUS treatment, suggesting an immediate reorganization of the cortico-muscular relationship after the tremor relief involving the cortical areas primarily related to the hand movement (27).

Measuring the out-strength from different frontoparietal areas directed toward the precentral area of the hemisphere receiving the Vim thalamotomy, we observed a significant decrease of cortico-cortical flow at T1, suggesting a reordering of cortico-cortical interactions and a reacquired “leadership” of the primary motor cortex recovering at T1, in coincidence with the tremor relief. The same is suggested by the significantly reduced out-strength deriving from the hemisphere ipsilateral with respect the MRgFUS target.

Changes found in cortico-cortical strength between areas not primarily involved in motor activity appear to be a further indicator of cortical reorganization. Changes in cortico-cortical and cortico-thalamic coupling in the beta band were already reported as excessive in Parkinson’s disease patients (37, 38), and suggested as a reliable measure of disease severity.

The reorganization of corticomuscular and cortico-cortical flow appears to a main factor resulting from thalamotomy. Interestingly it appears to be a “precondition” for the following outcome, even if all patients were substantially tremor-free at T1, suggesting that the achievement of a physiological reorganization is important for late prognosis.

The observation of high values of the clustering coefficient was rather noticeable and suggested an augmented tendency of different regions to express a pathological, not efficient, increase of connection between different nodes. This occurred in beta band, but also in low-gamma band. Using EEG signals, an increased local clustering coefficient was already noted in PD patients compared with healthy subjects (39), or the same patients in off with respect to on conditions (40). Similar evidence was obtained comparing PD with healthy subjects using MRI signals (41). This may suggest an increased cortical “pathological trafficking,” not limited to the involved motor areas, that is associated with the defective motor activity specific for PD patients and increases during motor activity. The values of the clustering coefficient decreased significantly at T1 both on the hemisphere Co to activated hand on the ipsilateral one. This finding also support the hypothesis that a pathological hyper-connectivity involving beta and low-gamma activity may thus act as a condition rather specific for PD, significantly attenuated after ViM lesion and predicting a better late outcome. In agreement, we did not find a similar connectivity pattern in patients with ET that we examined after MRgFUS in a similar way. We did not investigate frequencies higher than those included in the low gamma, so the involvement of these frequencies may just represent an “extension” of the results obtained in beta frequencies. In fact, the beta-low-gamma frequency range can be generated by the same neuronal systems (42).

Most of our results, including CMC, the out-strength from different cortical areas toward the motor area of the hemisphere with treated Vim (and contralateral to activated hand) mainly involved the beta band frequencies suggesting that the disordered network connecting different ROIs or local hyper-connectivity derive from the pathological organization of these frequencies. Actually, beta frequencies are typically involved in motor function both in healthy and pathological conditions, including PD patients (37, 43).

At rest, a condition in which the tremor had its maximum expression at T0, we did not identify significant relationships with the various indices relating to MEG frequencies, this could suggest that the theta rhythmicity of the tremor mainly involves the basal nuclei and reflects little on the cortical areas.

Regression analysis showed a significant relationship between the reduction of tremor-related scores evaluated at 1-year follow-up during motor activation and the reduction of out-strength from non-primary motor areas and precentral area ipsilateral to treated ViM observed at T1, the same was found between the reduced values of the clustering coefficient values measured in Co-SupM and Co-PostC ROI. This can suggest that excessive cortico-cortical flow is mainly disturbing motor activity, while increased clustering coefficient may influence the movement disorder both a rest and during action. The relationship between the Co-SupM and PostC areas with the primary motor area in PD is variably reported in the literature [see (44) for a review, (45)]. Even if our observation cannot resolve every single interpretation, it may however suggest that interactions and local organization of these areas play a significant role in motor impairment and possibly be “relieved” by ViM lesions.

This study had some limitations deriving from the small sample size and a higher number of patients who maintained a positive outcome 1 year after MRgFUS treatment, while only a few patients had a relevant recurrence of Parkinson’s symptoms. The slight number of unsuccessful MRgFUS treatments can be considered as positive result, but did not allow comparisons between groups with different prognosis. However, correlation analyses indicate a positive relationship between the evaluated network measure and the improvement maintained after 1 year of follow-up. This may suggest that the effort of identifying network changes may propose to verify in a more extensive case series of significant outcome-predicting factors.

Our results we obtained were rather clear, but obviously limited to not severe patients with prominently lateralized signs and dominant tremor. The validation of the parameters applied in a more complex series of patients with PD therefore requires further evaluations in order above all to confirm the possible predictive value of the effectiveness of the treatment.

## 5 Conclusion

Our data suggest that the higher cortico-cortical flow and pathological increased local connection between different nodes, revealed by the values of clustering coefficient, together with topographically disordered cortico-cortical flow and CMC may reveal an extensive and disarranged cortical defect occurring during motor activity.

The observation that most of the changes in the evaluated measures correlate with the changes in the clinical score related to the active movement may also suggest that MRgFUS Vim thalamotomy may act outside the resting tremor on the more complex motor impairment occurring in PD patients.

## Data availability statement

The data and MEG signals supporting the conclusions of this article will be made available by the authors on request.

## Ethics statement

The studies involving humans were approved by Fondazione IRCCS Istituto Neurologico Carlo Besta, Milan, Italy. The studies were conducted in accordance with the local legislation and institutional requirements. The participants provided their written informed consent to participate in this study. Written informed consent was obtained from the individual(s) for the publication of any potentially identifiable images or data included in this article.

## Author contributions

EV: Formal analysis, Investigation, Methodology, Validation, Writing – review & editing. FP: Conceptualization, Investigation, Methodology, Software, Validation, Writing – review & editing, Supervision. SF: Conceptualization, Formal analysis, Methodology, Supervision, Validation, Writing – original draft, Writing – review & editing. NG: Data curation, Investigation, Validation, Writing – review & editing, Methodology. RC: Data curation, Formal analysis, Validation, Writing – review & editing, Investigation. SR: Data curation, Validation, Writing – review & editing. DR: Data curation, Validation, Writing – review & editing. PL: Data curation, Resources, Validation, Writing – review & editing, Methodology. RE: Investigation, Project administration, Validation, Writing – review & editing.
